# Dental treatment approaches under general anesthesia in children with cancer

**DOI:** 10.4317/medoral.26902

**Published:** 2025-08-16

**Authors:** Esra Kızılcı, Kevser Kolcakoglu, Gul Yucel, Merve Kepezkaya

**Affiliations:** 1 Assoc Dr. DDS, PhD, Department of Pedodontics, Faculty of Dentistry, Erciyes University, Kayseri, Turkey; 2 M.D. Department of Pedodontics, Faculty of Dentistry, Pediatrician, Erciyes University, Kayseri, Turkey; 3 Research Assist, Department of Pedodontics, Faculty of Dentistry, Erciyes University, Kayseri, Turkey

## Abstract

**Background:**

To evaluate dental treatment approaches under general anesthesia in children with cancer.

**Material and Methods:**

DMF-T values of existing decay, missing and filled teeth of 68 pediatric patients receiving active cancer treatment were recorded. Systemic and physical examinations of patients were performed by a paediatrician. İntraoral and extraoral examinations of children were performed by pediatric dentists. The dental treatment plan encompasses the child's individualized oral health needs. Dental procedures are generally performed in the controlled environment of a hospital operating room under general anaesthesia. Analyzes were made with SPSS 25.0 package program.

**Results:**

The study determined that the average age was 6.47±2.93. In this study, extraction-focused treatments were used instead of restorative (r=0.346, *p*=0.01) and endodontic treatments (r=0.274, *p*=0.01).

**Conclusions:**

Despite the development of restorative and endodontic treatments under general anaesthesia, even pediatric crown applications, radical decisions must be made to control the medical condition of patients with childhood cancers.

** Key words:**Immunosuppression, dental care, general anesthesia, treatment protocol, child.

## Introduction

Childhood cancer continues to be the primary cause of disease-related mortality among children, spanning various age groups, ethnic backgrounds, and socioeconomic classes (1). Chemotherapeutic agents, radiation therapy, surgery, immunotherapy, and/or hematopoietic cell transplantation (HCT) may be used to treat the underlying malignancy in these children (2).

Immunosuppressive and/or radiation therapy can lead to various acute and long-term adverse effects in the oral cavity (3). Oral mucositis, associated pain, taste dysfunction, opportunistic infections (such as candidiasis, herpes simplex virus), dental caries, dry mouth (such as salivary gland dysfunction, xerostomia), neurotoxicity, mucosal fibrosis, gingival hypertrophy, osteoradionecrosis, and medical complications are just a few of the acute and chronic oral complications that may arise as a sequence of such therapies (3-5). Children receiving these therapies will experience positive outcomes from dental interventions that are timely, effective, and tailored to their medical history, cancer treatment plan, and overall health status (3). It is crucial to evaluate the condition of their oral cavity and address any dental complications (2). Dental care for these children can be complex, as their medical condition and ongoing treatments can have significant implications for their oral health. Each pediatric patient necessitates a personalized approach to their management. Hence, it is advisable to consult the patient's physicians and, if necessary, other dental specialists before initiating dental treatment (6). It is recommended to assess the child's neutrophil counts, platelet levels, absolute haemoglobin level (Hgb), and general health status before undertaking any dental procedure (3,7). These children usually receive cancer treatment in cycles or phases. Therefore, they require dental care under sedation or general anesthesia (3,7,8). Considering the aforementioned data, this study aimed to identify the variables that influence dental care, which is performed under general anaesthesia.

## Material and Methods

- Ethicals

Ethics approval for the study was provided by the Clinical Research Committee of the Erciyes University of Medicine (No:2023.785). Additionally, written informed consent was obtained from the parents of the participants. - Study design and participants

68 patients, who are receiving active cancer treatment and referred to the pediatric dentıstry department by the pediatric oncology service of the Children's Hospital for their oral health to be evaluated, were included in the study. Systemic and physical examinations of patients, who range in age from 2 to 14 years, were by conducted the paediatrician at the University Faculty of Dentistry Intraoral and extraoral examinations of the patients and oral condition were performed by specialization pediatric dentists at the University Faculty of Dentistry, Department of Pediatric Dentistry. The pediatric dentists possessed equivalent clinical experience and training. Panoramic radiographs were not obtained from these patients due to their compromised health condition. If deemed necessary, a periapical radiograph was obtained. The World Health Organization (WHO) has established standards for assessing dental caries, which involves quantifying the number of decayed, missing, and filled teeth (DMFT) (9) (SPSS Inc., Chicago, Illinois). The existing decayed, missing and filled teeth of these patients were evaluated.

- Treatment Planning

**Figure 1:**
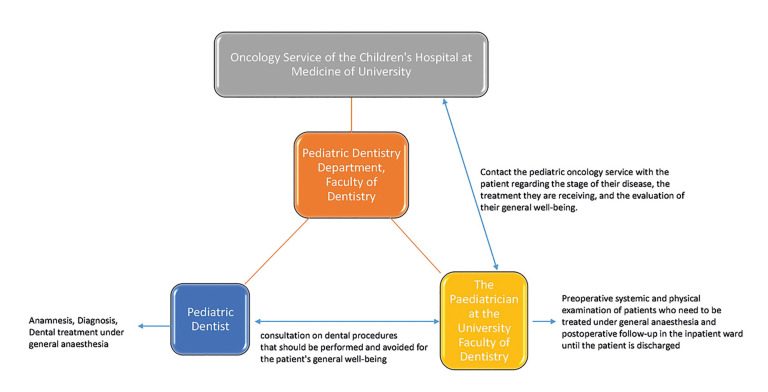
Dental treatment under general anesthesia workflow in children with cancer

The dental treatment plan encompasses the child's individualized oral health requirements. Dental procedures are typically conducted within the controlled environment of a hospital operating room, where general anesthesia is administered. The utilization of this particular conFiguration facilitates the meticulous observation of essential physiological indicators and prompt implementation of medical interventions, if deemed necessary. It is imperative that the administration of anesthesia be carried out by a proficient pediatric anesthesiologist possessing specialized knowledge in effectively managing cases involving complex medical conditions. It is imperative to prioritize the examination of potential drug interactions that may arise between anesthesia medications and cancer treatments (Fig. 1).

- Statistics

Descriptive statistics are presented with frequency, percentage, median, min and max values. In the study, the Mann-Whitney U test and the Kruskall Wallis test were used to examine the measurements according to the groups. The chi-square test was used to examine patient characteristics in diagnostic groups. The spearman correlation test was used to examine the relationship between the number of anesthesia, extraction, filling and caries numbers. *P* values less than 0.05 were considered statistically significant in the study. Analyzes were made with SPSS 25.0 (SPSS Inc., Chicago, Illinois) package program.

## Results

The study findings revealed that 67.6% of the participants were identified as male, while 32.4% were identified as female. The patients were diagnosed with various medical conditions, including Acute Lymphoblastic Leukemia (ALL) accounting for 32.4% of cases, Neuroblastoma accounting for 11.8%, Chronic Granulomatosis accounting for 5.9%, and Acute Myeloid Leukemia (AML), Aplastic Anemia, and Mucopolysaccharidosis Type 1 each accounting for 4.4% of cases. Upon examination of the patient groups, it was ascertained that 55.9% of the individuals belonged to the primary dentition group, while 7.4% were categorized under the permanent dentition group (Table 1).

The analysis revealed that there was no significant difference in the gender distribution across the groups. There was 26 male (68.4%) and 12 female (31.6%) in primary dentition. There was 16 male (64.0%) and 9 female (36.0%) in mixed dentition. There was 4 male (80.0%) and 1 female (20.0%) in permanent dentition. There was no significant difference in gender distribution among the patients in the primary, mixed, and permanent dentition groups (*p*=0.38, *p*>0.05).

The study determined that the average age was 6.47±2.93. It was found that the ages varied across the different groups. The discrepancy in age distribution between the primary dentition group and the mixed and permanent groups was found to be statistically significant (*p*=0.01, *p*<0.05) (Table 2).

In Table 2, the study findings indicate that there was no significant difference in the number of initial caries among the groups (*p*=0.92, *p*>0.05). The study revealed that there were varying levels of extracted teeth among the groups at the initial stage. The observed discrepancy can be attributed to a reduced count of extracted teeth in the primary dentition group (*p*=0.04, *p*<0.05). It was observed that there were varying levels of fillings at the initial stage among the groups. The observed disparity in the number of extracted teeth at the baseline level between the permanent group and the control group was found to be statistically significant (*p*=0.03, *p*<0.05). The study findings indicate that there was no significant difference in the number of caries at the post-treatment level among the groups (*p*=0.99, *p*>0.05). The study findings indicate that there was no significant difference in the number of shots at the post-treatment level between the groups (*p*=0.07, *p*>0.05). The study findings indicate that there was no significant difference in the number of fillings at the post-treatment level among the groups (*p*=0.49, *p*>0.05). It was found that the levels of general anaesthesia in the procedures varied among the groups. The observed disparity can be attributed to the fact that the Permanent group received a significantly greater amount of anaesthesia during the baseline procedure (*p*=0.02, *p*<0.05).

The study revealed that the initial caries levels varied depending on the type of disease. The observed disparity can be attributed to the elevated prevalence of caries in patients with acute myeloid leukaemia (AML) (*p*=0.01, *p*<0.05). The study revealed variations in the number of initial shots based on the specific type of disease. The observed disparity can be attributed to the increased administration of Chronic granulomatosis shots (*p*=0.03, *p*<0.05). The analysis revealed that there was no significant difference in the number of general anaesthesia administered across different disease types (*p*=0.18, *p*>0.05) (Table 3).

In Table 4, the study observed a positive and modest correlation (r=0.346, *p*=0.01) between the initial number of caries and the subsequent number of extractions following treatment. The study revealed a significant positive correlation (r=0.686, *p*=0.01) between the initial number of caries and the subsequent number of fillings following treatment. The study revealed that individuals with higher initial levels of caries received a greater number of fillings, while those with lower levels were more likely to undergo tooth extraction. The study revealed a positive and weak correlation (r=0.274, *p*=0.01) between the initial number of fillings and the final number of shots administered during the treatment. The study findings indicate that there was no statistically significant correlation between the initial number of fillings and the final number of fillings after treatment (r=0.231, *p*=0.06).

## Discussion

Oral health care is imperative for children who have been diagnosed with cancer. Oral complications such as infections should be anticipated during cancer treatment1. In the present study, as in other studies, the authors have examined the approach to dental treatment in children with cancer therapy (1,2,4).

Every year, over 40,000 children receive cancer treatment. Leukemias are the most common type of cancer, followed by tumors of the brain and other central nervous systems, sarcomas, neuroblastomas, and renal tumors(1,10). A small percentage of childhood cancer cases are linked to genetic syndromes like Down syndrome and environmental factors like previous ionizing radiation exposure (10-13). Furthermore, children with immunodeficiencies are more likely to develop certain cancers, most notably non-Hodgkin lymphoma and Kaposi sarcoma (14). In this study, the most common acute lymphoblastic leukemia was found in children, while immunodeficiency disorders were less common.

Because presecretory odontoblasts divide rapidly during odontogenesis in children, they are especially vulnerable to the harmful effects of alkylating agents or radiation. The formation and growth of enamel crystals, which obtain calcium and phosphorus ions from calcified collagen fibrils, follows the sequential events of dentin calcification (15). Primary tooth germs are rarely damaged during cancer therapy because their formation begins in utero and continues for the next 3 to 4 years (15,16). Permanent teeth, on the other hand, develop hard tissue early in life (16). Few studies have looked at the condition of cancer survivors' deciduous dentition, as most papers focus on permanent dentition. According to two independent studies, patients who had neuroblastoma had higher caries scores in primary dentition (15,17). The majority of the children with cancer survivors in this study were in their primary dentition (*p*=0.01, *p*<0.05). Due to children who received cancer treatment at an earlier age, this result was expected by the authors.

Proc *et al*. indicated that DMFT was significantly higher in children with cancer survivors. This is in accordance with the papers, which demonstrated a relationship between cancer therapy and the incidence of caries. Duggal *et al*. found a relationship between the cancer therapy of children and their siblings are similar DMFT scores. In the current study, it has been determined that the number of caries is not significant in different dentitions, while the caries percentage is higher in permanent dentition. (*p*=0.92>0.05).

Radiotherapy and/or chemotherapy are usually the treatments chosen for common types of pediatric cancer. The type of drug chosen and the dose of radiotherapy may cause dental anomalies. It has been stated that the 4 Gy dose of radiotherapy causes the onset of harmful effects on dentition (15). In addition, a dose of 24 Gy increases the incidence of dental caries (1,15). Most children who received long-term cancer treatment had a higher number of caries (10).

Most children who received long-term cancer treatment had a higher number of cavities (18). In this study, although ALL patients were more numerous, AML patients were more likely to have caries (*p*=0.01, *p*<0.05). This may be due to the course of the disease and the individualization approach of radiotherapy and/or chemotherapy (1).

Blood and biochemistry values may change at any time in children receiving cancer treatment. It is often preferred to treat these children while their systemic values are under control (3). In addition, since these children are frequently exposed to repeated injection procedures during medical treatment, it is necessary to prevent and alleviate procedure-related distress (19). Furthermore, restorations performed under general anaesthesia appear to have better marginal adaptation and less secondary caries than those performed under sedation or clinic (20). For this reason, general anesthesia, which allows multiple dental treatments, is preferred (8). In this context, dental approaches for children with cancer treated under general anesthesia for oral rehabilitation require a comprehensive and individualized approach (1). The primary focus is on providing safe and effective dental care while considering the child's unique medical condition, cancer treatment, and overall well-being. Collaboration among the medical and dental teams is key to achieving the best possible outcomes for these young patients (3,8). Although general anesthesia has the advantage of providing oral rehabilitation in one-stage, it may lose this advantage in children with cancer who receive complex treatment (21). Almeida *et al*. stated that 79% of children treated under general anesthesia had new carious lesions two years later (22). Therefore, more than one general anesthesia can be performed in children (21). In this study, authors have evaluated the number of general anaesthesia for dental treatment and different childhood cancers. No relationship was found between the number of general anesthesia and the types of diseases (*p*=0.18, *p*>0.05).

Pediatric dentısts have to make decisions about the details of dental treatment, such as the choice of restorative materials and treatment techniques, knowing that the child is likely to be immunosuppressed soon. Ideally, all dental treatment should be completed prior to commencement of cancer treatment (7,23,24). Teeth that are clinically and radiographically sound should not be extracted (2). Teeth with exfoliative stages should be allowed to naturally. Infected teeth, nonrestorable teeth, infected root apex, and periodontally compromised teeth should be extracted 1-2 weeks prior to initiation of cancer therapy to allow time for healing (2,7,24). In this study, extraction-focused treatments were used instead of restorative (r=0.346, *p*=0.01) and endodontic treatments (r=0.274, *p*=0.01).

- Limitations

The patients were not cooperative due to their medical conditions. Periodontal status could not be evaluated. Space maintainers could not be planned for any patient due to the possibility of periodontal disease.

## Conclusions

Despite the development of restorative and endodontic treatments under general anaesthesia, even pediatric crown applications, radical decisions must be made to control the medical condition of patients with childhood cancers.

## Figures and Tables

**Table 1:** Demographic variables.

Demographic Variables		N (Number)	%(Percentage)
Gender	Male	46	67.6%
	Female	22	32.4%
Diagnosis	Acute lymphoblastic leukaemia (ALL)	22	32.4%
	Neuroblastoma	8	11.8%
	Acute myeloid leukaemia (AML)	4	5.9%
	Chronic granulomatosis	4	5.9%
	Hodking lymphoma	4	5.9%
	Aplastic anemia	4	5.9%
	Mucopolysaccharidosis type1	3	4.4%
	Combined immunodeficiency	2	2.9%
	Thalassemia major	2	2.9%
	B-cell ALL	2	2.9%
	Osteopetrosis	1	1.5%
	Medulloblastoma	1	1.5%
	Adrenoleukodystrophy	1	1.5%
	Juvenile myelomonocytic leukemia	1	1.5%
	Alveolar rhabdomyosarcoma	1	1.5%
	Platelet failure	1	1.5%
	Hemophagocytic lymphohistiocytosis	1	1.5%
	İmmunodeficiency with ıge syndrome	1	1.5%
	Immunodeficiency with DOCK8 mutation	1	1.5%
	Fanconi anemia	1	1.5%
	CD40 Deficiency immunodeficiency	1	1.5%
	Pleuropulmonary blastoma	1	1.5%
	İatrogenic immunodeficiency	1	1.5%
Groups	Primary dentition	38	55.9%
	Mixed dentition	25	36.8%
	Permanent dentition	5	7.4%

**Table 2:** Examination of general characteristics by groups and the number of decays, missing, filling.

	Groups							
	Primary Dentition^1^		Mixed Dentition^2^		Permanent Dentition^3^		p	
	µ	min-max	µ	min-max	µ	min-max				
Age	4.5	2-6	9	5 -11	13	12 -14	0.01*	-
Before treatment Decay	8.5	1-20	2	20-9	9	3-18	0.92	-
Before treatment Missing	0	0-7	1	8-0	1	0-7	0.04*	1<2.3
Before treatment Filling	0	0-6	0	6-2	2	0-2	0.03*	3>1.2
After treatment Decay	0	0-0	0	0-0	0	0-0	0.99	-
After treatment Missing	2	0-14	0	13-1	1	0-7	0.07	-
After treatment Filling	8	0-13	0	19-11	11	5-20	0.49	-
How many anesthesia procedures could be performed?	1	1-5	1	4-2	2	1-6	0.02*	3>1.2

*0.05 statistical significance.

**Table 3:** Number of decayed and extracted teeth. number of anesthesia by disease in before treatment.

Disease	Before Treatment Decay	Before Treatment Missing	How Many Anesthesia Procedures Could Be Performed?
	µ (Min-Max)	µ (Min-Max)	µ (Min-Max)
All	8 (2-20)	0(0-8)	1(1-6)
Neuroblastoma	8.5(1-16)	0(0-0)	1(1-1)
Aml	14(8-20)*	0(0-5)	1.5(1-3)
Chronic granulomatosis	9(4-12)*	2(0-5)*	1.5(1-5)
Hodgkin lymphoma	9(4-18)	0(0-7)	1.5(1-4)
Aplastic anemia	7.5(5-11)	0(0-4)	1.5(1-3)
Mucopolysaccharidosis type1	7(2-10)	0(0-0)	1 (1-2)
p	0.01*	0.03*	0.18

**Groups with n>3 were included *Significant difference at 0.05 level.

**Table 4:** Examination of the number of decays. extracted teeth and fillings.

		Before Treatment Decay	Before Treatment Missing	Before Treatment Filling	After Treatment Decay	After Treatment Missing	After Treatment Filling
Before Treatment Decay	r	1.00	-	-	-	-	-
	p	-	-	-	-	-	-
Before Treatment Missing	r	-0.05	1.00	-	-	-	-
	p	0.67	-	-	-	-	-
Before Treatment Filling	r	-0.20	.722^*^	1.00	-	-	-
	p	0.11	0.00	-	-	-	-
After Treatment Decay	r	0.01	0.01	0.01	0.01	-	-
	p	1.00	1.00	1.00	1.00	-	-
After Treatment Missing	r	.346^*^	.564^*^	.274^*^	0.01	1.00	-
	p	0.00	0.00	0.02	1.00	-	-
After Treatment Filling	r	.686^*^	0.16	0.231	0.01	-0.01	1.00
	p	0.00	0.20	0.06	1.00	0.96	-

*0.05 statistical significance.
